# Long-Term Angiotensin II Infusion Induces Oxidative and Endoplasmic Reticulum Stress and Modulates Na^+^ Transporters Through the Nephron

**DOI:** 10.3389/fphys.2021.642752

**Published:** 2021-04-01

**Authors:** Bruna Bezerra Lins, Fernando Augusto Malavazzi Casare, Flávia Ferreira Fontenele, Guilherme Lopes Gonçalves, Maria Oliveira-Souza

**Affiliations:** Laboratory of Renal Physiology, Department of Physiology and Biophysics, Institute of Biomedical Sciences, University of São Paulo, São Paulo, Brazil

**Keywords:** angiotensin II, oxidative stress, endoplasmic reticulum stress, inflammation, kidney sodium transporters

## Abstract

High plasma angiotensin II (Ang II) levels are related to many diseases, including hypertension, and chronic kidney diseases (CKDs). Here, we investigated the relationship among prolonged Ang II infusion/AT1 receptor (AT1R) activation, oxidative stress, and endoplasmic reticulum (ER) stress in kidney tissue. In addition, we explored the chronic effects of Ang II on tubular Na^+^ transport mechanisms. Male Wistar rats were subjected to sham surgery as a control or prolonged Ang II treatment (200 ng⋅kg^–1^⋅min^–1^, 42 days) with or without losartan (10 mg⋅kg^–1^⋅day^–1^) for 14 days. Ang II/AT1R induced hypertension with a systolic blood pressure of 173.0 ± 20 mmHg (mmHg, *n* = 9) compared with 108.0 ± 7 mmHg (mmHg, *n* = 7) in sham animals. Under these conditions, gene and protein expression levels were evaluated. Prolonged Ang II administration/AT1R activation induced oxidative stress and ER stress with increased Nox2, Nox4, *Cyba* and *Ncf1* mRNA expression, phosphorylated PERK and eIF2α protein expression as well as *Atf4* mRNA expression. Ang II/AT1R also raised *Il1b, Nfkb1* and *Acta2* mRNA expression, suggesting proinflammatory, and profibrotic effects. Regarding Na^+^ tubular handling, Ang II/AT1R enhanced cortical non-phosphorylated and phospho/S552/NHE3, NHE1, ENaC β, NKCC2, and NCC protein expression. Our results also highlight the therapeutic potential of losartan, which goes beyond the antihypertensive effect, playing an important role in kidney tissue. This treatment reduced oxidative stress and ER stress signals and recovered relevant parameters of the maintenance of renal function, preventing the progression of Ang II-induced CKD.

## Introduction

The renin-angiotensin system (RAS) activity triggers the octapeptide angiotensin II (Ang II) synthesis, which exerts its effects by binding to two distinct G-protein-coupled receptors, AT1 (AT1R) and AT2 (AT2R; Gubler and Antignac, 2010). Under physiological conditions, Ang II controls extracellular volume, vascular tone and cardiac function in both humans and rodents. However, high plasma levels of Ang II are often related to hypertension, renal damage and cardiac fibrosis. In this context, AT1R antagonists are known for their therapeutic effects on cardiovascular and renal disorders, as well diabetes (Villapol and Saavedra, 2015).

The exposure of rats to Ang II (270 ng⋅kg^–1^⋅min^–1^), even short-term treatment (1 h), resulted in rapid smooth muscle contraction, a pressor response, and consequent acute kidney vasculature and tubulointerstitial damage (Romero and Reckelhoff, 1999). In addition, extended Ang II infusion from 1 to 2 weeks resulted in hypertension, enhanced reactive oxygen species (ROS) production (Nishiyama et al., 2001), increased preglomerular vascular resistance accompanied by inward hypertrophic remodeling and a reduced glomerular filtration rate (GFR; Stevenson et al., 2000; Edgley et al., 2003). In a recent study, we showed that Ang II (200 ng⋅kg^–1^⋅min^–1^) infusion over 2 weeks, which activated AT1R, induced severe preglomerular vascular injury, decreased renal vascular resistance, and increased renal plasma flow (RPF) and GFR, consistent with outward preglomerular remodeling, as well as albuminuria, glomerulosclerosis, and natriuresis (Casare et al., 2016). In spite of this, Ang II involvement on oxidative stress, endoplasmic reticulum (ER) stress or tubular Na^+^ transporters were not investigated.

Oxidative stress is related to an imbalance in ROS production. ROS (or other oxidants) are essentially composed by superoxide anions, hydrogen peroxide, and hydroxyl radicals (Pieczenik and Neustadt, 2007), which are produced by the metabolism of living organisms and are eliminated by antioxidant defensive mechanisms (Birben et al., 2012). Under physiological conditions, ROS also can regulate intracellular signal transduction pathways (Daenen et al., 2019). The excessive production of ROS results in oxidation of various compounds, including lipids, proteins, and DNA, and consequent metabolic disorders due to the inactivation of essential factors associated with cellular function (Di Meo et al., 2016). Moreover, many sources of ROS, including the nicotinamide dinucleotide (phosphate) NAD(P)H oxidase enzyme complex (or NADPH oxidases), which consists of Nox1, Nox2 (gp91phox), Nox3, Nox4, Nox5, Duox1, and Duox2, are present in different tissues (Kawahara et al., 2007). In chronic kidney diseases (CKDs), the Nox1, Nox2, Nox4, and Nox5 isoforms (Jones et al., 1995) are important sources of ROS in kidney tissue (Muñoz et al., 2020). The Nox2 isoform requires the regulatory subunit p22*^*phox*^* and the cytosolic organizer subunit p47*^*phox*^* to participate on tubular electrolyte transport (Sedeek et al., 2013). Nox4 requires interactions with p22*^*phox*^* but not p47*^*phox*^* (Bedard et al., 2007), and its activity seems to be involved in fibroblast-to-myofibroblast differentiation, which results in kidney fibrosis, an associated condition with diabetes and hypertension (Gorin et al., 2005; Bondi et al., 2010). In addition, accumulating evidence has revealed that oxidative stress and ER stress potentiate each other in several diseases. Indeed, a positive feedback loop suggests that NOX-mediated oxidative stress activates ER stress, which subsequently induces NOX activity (Li et al., 2010). Although both Nox2 and Nox4 appear to be implicated in kidney disease (Sedeek et al., 2013), the contributions of these enzymes to extended Ang II/AT1R-induced oxidative stress, ER stress and/or chronic kidney damage have not been fully elucidated.

The kidneys play a central role in the long-term control of blood pressure by regulating sodium balance and extracellular fluid volume. However, Ang II at high plasma levels serves as a stimulus for fluid and electrolyte reabsorption, resulting in hypertension (Nguyen et al., 2015). Interestingly, the time and dose of Ang II infusion on experimental models are relevant factors that not only modulate the progression of renal diseases associated with hypertension but also regulate kidney Na^+^ transporters, although many of Ang II effects on kidney function have typically been evaluated for 1 or 2 weeks (Tiwari et al., 2009, 2010; Nguyen et al., 2015).

Considering our previous findings, in which Ang II infusion over 2 weeks and AT1R-mediated effects were related to significant kidney function loss (Casare et al., 2016), we were encouraged to investigate the factors involved in kidney injury using this experimental model. Furthermore, numerous studies have been reported that AT1R antagonists, including losartan and valsartan, mainly affect systemic and renal hemodynamics (Siragy et al., 2002), as well as the inflammatory, fibrotic and oxidative stress responses of cardiac tissue (Wen et al., 2012, 2013). Thus, the present study aimed to further investigate the contribution of extended Ang II exposure and AT1R activity to oxidative stress and ER stress, proinflammatory and profibrotic factors, and tubular Na^+^ transporters expression and distribution on kidney tissue.

Collectively, our results support a model in which high levels of circulating Ang II contribute to sustained hypertension, oxidative and ER stress associated with inflammation and fibrosis leading to CKD progression, as well as relevant changes on renal Na^+^ transport. This study also highlights the therapeutic potential of losartan in preventing the progression of Ang II-induced CKD.

## Materials and Methods

### Animals

Thirty-three male Wistar rats aged 60 days and weighing between 150–200 g were purchased from the animal care facility of the Institute of Biomedical Sciences, the University of São Paulo (São Paulo, Brazil) and were housed at the facility of the Department of Physiology and Biophysics of the same institute. All animals were maintained under a 12:12-h light-dark cycle with free access to water and food. The protocols were conducted in accordance with the Ethics Committee on the Use of Experimental Animals of the University of São Paulo (No. 37/2016) and were in accordance with the ethical principles adopted by the Brazilian Society of Laboratory Animal Science. The animal groups and experimental procedures were previously described by Casare et al. (2016) and are summarized here. The animals were acclimated for 5 days, and at 65 days of age, they were randomly divided into four groups: sham rats (with sham surgery), losartan-treated rats, Ang II-treated rats, and Ang II- and losartan-treated rats.

The animals were anesthetized with ketamine (75 mg/kg, i.p.) and xylazine (4 mg/kg, i.p.; Virbac, Jurubatuba, São Paulo, Brazil) and immediately placed on a hot plate to maintain a constant temperature at 37°C, and after total loss of pain reflexes, a dorsal midline incision was made to create a subcutaneous (s.c.) pocket, where the osmotic minipump (model 2006, Alzet Osmotic Pumps Company, Cupertino, CA, United States) containing Ang II (200 ng/kg/min, Tocris Bioscience, Bristol, United Kingdom, diluted in 0.9% NaCl solution) was inserted for continuous infusion of Ang II for 42 days. For all animals, after surgery, the skin was sutured, and asepsis was performed. The sham group was also observed for the same time period after sham surgery and received daily injection of 200 μL/100 g of 0.9% NaCl solution (s.c.). The Ang II/losartan group was cotreated with losartan (10 mg/kg/day, s.c., DuPont 753, Merck Pharmaceuticals, Deepwater, NJ, United States, diluted in 0.9% NaCl solution) between the 28th and 42nd days after Ang II minipump insertion. A group of rats was treated only with losartan [10 mg/kg/day, diluted in 0.9% NaCl solution (s.c.)] and used as a control for the Ang II/losartan group. At the end of the treatment, the rats were placed individually in metabolic cages (Techniplast, Milan, Italy) for 24 h, and body weight (BW), food (g/day) and water (mL/day) intake and urine output (μL/min) were evaluated. BW gain was calculated using the following equation: (final BW – initial BW)/6 weeks.

### Systolic Blood Pressure

Changes in blood pressure were evaluated using non-invasive tail-cuff plethysmography (Panlab/Harvard Apparatus, Barcelona, Spain), as previously described (Thieme and Oliveira-Souza, 2015) and are summarized here. The rats were acclimatized to the apparatus for 20 min before the blood pressure measurements. Then, the tail artery was dilated by placing the animal in a thermostatically controlled plastic holder that was heated for 20 min. The pulse was detected by passing the tail through a tail-cuff sensor that was attached to the amplifier. Systolic blood pressure (SBP) measurements were considered to be successful if the rat did not move, and a clear initial and constant pulse could be detected. The average values for the SBP were subsequently obtained from eight sequential cuff inflation-deflation cycles.

### Plasma and Urinary Parameters

At the end of treatment, the animals were anesthetized as described above, and after the complete loss of pain reflexes, they were placed on a warm table to maintain body temperature, prepared surgically for cannulation of the distal aorta using a PE-50 catheter (Clay Adams Company, Inc., Parsippany, NJ, United States) for blood sample collection and then euthanized by exsanguination. Immediately, an abdominal incision was made using a scalpel, and the urinary content of the bladder was collected. Next, the left kidney was removed, weighed, and cut into two sections. One section was quickly frozen and pulverized in liquid nitrogen for further analysis by RT-PCR. For the remaining section, the cortex and medulla were mechanically separated using a scalpel, and each section was crushed in PBS solution (0.15 M NaCl containing 10 mM sodium phosphate buffer, pH 7.4) with protease inhibitors (Merck Pharmaceuticals), centrifuged (4,000 × *g* for 10 min at 4°C) and frozen at −80°C for protein analysis by Western blotting. The right kidney was perfused with PBS solution, removed, cross-sectioned, inserted into properly labeled histological cassettes and fixed in 4% paraformaldehyde solution. After dehydration, the slices were embedded in paraffin for morphological studies as previously described (de Ponte et al., 2017). Plasma osmolality was evaluated using an osmometer (Wescor, Logan, UT, United States), and the plasma sodium concentration was measured by flame photometry (9180 Electrolyte Analyzer; Roche, Basel, Switzerland) as previously described (Casare et al., 2016; de Ponte et al., 2017).

### mRNA Expression

As previously described (Casare et al., 2016; de Ponte et al., 2017) and summarized here, frozen kidney sections were homogenized in liquid nitrogen and then resuspended in TRIzol LS Reagent (Invitrogen, Carlsbad, CA, United States) for RNA extraction (GE Healthcare Life Sciences, BW, Germany) according to the manufacturer’s instructions. Two hundred nanograms of RNA was used for the synthesis of cDNA (RT-PCR) with a High-Capacity RNA-to-cDNA kit (Applied Biosystems, Foster City, CA, United States) and a Veriti Thermocycler (applied biosystems, Foster, CA, United States), according to the product protocols. The obtained cDNA was used for the analysis of gene expression through quantitative real-time polymerase chain reaction (qPCR; StepOnePlus Real Time PCR System, Applied Biosystems). The following FAM-labeled probes obtained in the TaqMan Gene Expression Assay format (Applied Biosystems) were used: NADPH oxidase isoform 4 (Nox4), Rn00585380_m1; NADPH oxidase isoform 2 (Nox2), Rn00576710_m1; NADPH oxidase subunit p22*^*phox*^* (*Cyba*), Rn00577357_m1; NADPH oxidase subunit p47*^*phox*^* (*Ncf1*), Rn00586945_m1; activating transcription factor 4 (*Atf4*), Rn00824644_g1; nuclear factor kappa B 1 (*Nfkb1*), Rn01399572_m1; interleukin 1 beta (*Il1b*), Rn00580432_m1; α-SMA (*Acta2*), Rn01759928_g1; and glyceraldehyde-3-phosphate dehydrogenase (*Gapdh*), Rn01775763_g1 (reference gene). Real-time PCR reactions were performed in duplicate, and the data were analyzed by the comparative cycle threshold (2^ΔΔ*Ct*^) method. The results were normalized to *Gapdh* expression and are shown as units relative to the control group.

### Immunoblotting

Frozen renal cortex samples were prepared for protein expression analyses as previously described (Costa-Pessoa et al., 2014; Gonçalves et al., 2018). Immunoblotting analysis was performed on 50-μg protein aliquots resolved by 9–15% SDS-PAGE. Then, the protein samples were transferred to polyvinylidene fluoride (PVDF) membranes (GE Healthcare Life Sciences). The membranes were incubated with the following primary antibodies: rabbit monoclonal anti-PERK [1:3000, #3192 (C33E10), Cell Signaling, reactivity H, M, and R]; rabbit monoclonal anti-phospho-Thr980 PERK (1:5000, #G.305.4, Thermo Fisher, Rockford, United States, reactivity M and R); rabbit monoclonal anti- eIF2α [1:1000, #5324S (D7D3), Cell Signaling, reactivity H, M, R, and Mk]; rabbit monoclonal anti-phospho Ser51 eIF2α [1:1000, #3597 (119A11), Cell Signaling, reactivity H, M, R, Mk, and Dm]; rabbit polyclonal anti-α-SMA (1:1500, #ab5694, Abcam, Cambridge, United Kingdom, reactivity H and M); rabbit polyclonal anti-NHE3 (1:1000, #SPC400D, StressMarq, Victoria, BC, Canada, reactivity H and R); mouse monoclonal anti-phospho-Ser552 NHE3 Clone 14D5 [1:3000, #MABN2415, (Sigma Aldrich, St. Louis, MO, United States), reactivity M, H, and R]; rabbit polyclonal anti-NCC (1:5000, #GTX41969, GeneTex, Irvine, CA, United States, reactivity H, M, and R); rabbit polyclonal anti-NKCC2 (1:1000, #38436, Cell Signaling, reactivity H, M, and R); rabbit polyclonal anti-NHE1 (1:2000, #ab67313, Abcam, reactivity R and H) and rabbit polyclonal anti-ENaC β (1:1000, SPC-404D, reactivity H, M, and R). A mouse monoclonal anti-β-actin antibody (1:5000, #ab170325, Abcam, Cambridge, United Kingdom, reactivity H, M, R, and Rb) was used as an endogenous control. Then, the membranes were incubated with conjugated goat secondary antibodies against rabbit or mice (#111-035-144 and 115-035-146, respectively, Jackson ImmunoResearch Laboratories, Baltimore, MD, United States). After that, the membranes were washed and exposed to the reagent for immunodetection with ECLTM luminescence (Amersham, GE) and subsequently to an imaging system (Amersham Imager 600, GE). The intensity of the bands was assessed by optical densitometry using ImageJ software (National Institute of Health). Protein expression was quantified relative to the expression of β-actin (loading control), and the results are presented as protein expression relative units to the control group. All antibodies were validated by the manufacturer’s information through datasheets.

### Immunofluorescence

The immunofluorescence experimental protocol was previously described by Cardoso et al. (2018) and summarized here. Fixed 4-μm kidney sections were deparaffinized and blocked with 3% BSA in PBS for 1 h at room temperature before being incubated overnight at 4°C with rabbit polyclonal anti-NCC (1:200, #GTX41969, GeneTex, Irvine, CA, United States, reactivity H, M, and R), rabbit polyclonal anti-NKCC2 (1:200, #38436, Cell Signaling, reactivity H, M, and R), and mouse monoclonal anti-α-1 Na^+^K^+^ pump (1:300, #05369, Clone C4646 Millipore, Burlington, MA, United States, reactivity H, R, M Rb, and Mk). Next, the kidney sections were incubated with Alexa Fluor 594-conjugated F(ab‘) goat anti-rabbit (1:200, #A11072) and Alexa Fluor 488-conjugated F(ab‘) rabbit anti-goat (1:200, #A21222) secondary antibodies (Thermo Fisher Scientific) for 1 h at room temperature in the dark and mounted with Fluoroshield (Sigma Aldrich). Protein staining was analyzed using a Zeiss LSM 510 confocal microscope equipped with a 40 × objective and laser excitation at 488 or 543 nm. The numbers of positive tubules were evaluated in five areas per kidney, and the mean relative fluorescence intensities of the control and treated groups were compared. The immunofluorescence analyses were performed blindly by one independent investigator, and the antibodies were validated by the manufacturer’s information through datasheets.

### Statistical Analysis

The results presented as the mean ± S.D. were analyzed by two-way analysis of variance (ANOVA) using GraphPad Prism 8.0 software (GraphPad Software, Inc., San Diego, CA, United States). We analyzed the interaction between the two factors (Ang II and losartan), and when the interaction was statistically significant, a Bonferroni *post hoc* test was performed for multiple comparisons. *p* < 0.05 was considered to indicate a statistically significant difference. The effect of Ang II *vs* the sham group was also assessed by multiple comparisons tests.

## Results

### Physiological Parameters and Blood Pressure

As shown in Table 1, BW gain [(final BW – initial BW)/6 weeks] and kidney weight were similar in all groups and consistent with previous results of our group using the same experimental model (Casare et al., 2016). Before the treatment, SBP was similar in all groups. At the end of the treatment, the sham and losartan-treated animals did not exhibit changes in SBP. However, after prolonged Ang II treatment, the rats displayed significant increases in SBP compared to the sham group rats, as shown by multiple comparisons tests. Two-way ANOVA showed that losartan cotreatment reduced SBP in the Ang II-treated rats (interaction *p* = 0.0001). Plasma Na^+^ concentrations and osmolality did not exhibit significant changes between the groups.

**TABLE 1:** Physiological parameters and blood pressure.

		Multiple	Comparisons		Two-way ANOVA Interactions
Parameters	Sham (6–7)	AII (7–9)	Los (6–9)	AII/Los (5–8)	AII/Los
Initial SBP, mmHg	100 ± 7	101 ± 8	100 ± 10	103 ± 10	*F* (1, 29) = 0.1149^ ns^
					*p* = *0.7371*
Final SBP, mmHg	108 ± 7	173 ± 20****	112 ± 8	110 ± 6^####^	*F* (1, 29) = 61.51^++++^
		*p* = *0.0001*	*p* > *0.9999*	*p* > *0.0001*	*p* = *0.0001*
Final body wg, g	418 ± 41	423 ± 20	442 ± 28	435 ± 28	*F* (1, 29) = 0.2858^ ns^
		*p* = *0.8348*	*p* > *0.9999*	*p* > *0.9999*	*p* = *0.5970*
Body wg gain, g/6 week	40.3 ± 3.0	39.1 ± 3.9	39.5 ± 6.1	33.0 ± 8.9	*F* (1, 29) = 1.562^ ns^
		*p* > *0.9999*	*p* > *0.9999*	*p* > *0.2737*	*p* = *0.2213*
Kidney wg/body wg, mg/g	3.88 ± 0.49	4.08 ± 0.44	4.11 ± 0.65	4.18 ± 0.66	*F* (1, 22) = 0.8010^ ns^
		*p* > *0.9999*	*p* > *0.9999*	*p* > *0.9999*	*p* = *0.7797*
Urine flow, μL/min	29.7 ± 4.9	44.6 ± 5.7**	31.9 ± 5.9	32.6 ± 11.2^#^	*F* (1, 29) = 7.840^++^
		*p* = *0.0020*	*p* > *0.9999*	*P* = *0.0127*	*p* = *0.0090*
Urine Na^+^, mEq/L	35.2 ± 4.2	32.9 ± 10.3	29.4 ± 4.5	37.7 ± 7.7	*F* (1, 23) = 3.539^ ns^
		*p* > *0.9999*	*p* = *0.9835*	*p* > *0.9999*	*p* = *0.0726*
Plasma Na^+^, mEq/L	141.0 ± 5	138.0 ± 3	139 ± 3	141.0 ± 3	*F* (1, 21) = 2.89^ ns^
		*p* = *0.6097*	*p* > *0.9999*	*p* = *0.8195*	*p* = *0.1038*
Plasma osmolality, mOsm/kg. H_2_O	296.0 ± 7	310.0 ± 7	305.0 ± 6	301.0 ± 2	*F* (1,20) = 3.92^ ns^
		*p* = *0.0717*	*p* = *0.6064*	*p* > *0.9999*	*p* = *0.0617*

Two-way ANOVA with Bonferroni multiple comparisons test was performed to compare data (means ± SD). The number of animals per group is indicated in parentheses. For multiple comparisons: **p < 0.01, ****p < 0.0001 *vs* sham (control); ^#^*p* < 0.05 and ^####^*p* < 0.0001 *vs* AII. *F* values and significance of the two-way ANOVA considering the treatments with angiotensin AII (AII), Losartan (Los), and their interactions were given. ns and ^+^indicate non-significant and significant at ^++^*p* < 0.01 ^++++^*p* < 0.0001, respectively. *p, p*-value. wg, weight.

### Oxidative Stress-Related Gene Expression

**FIGURE 1:**
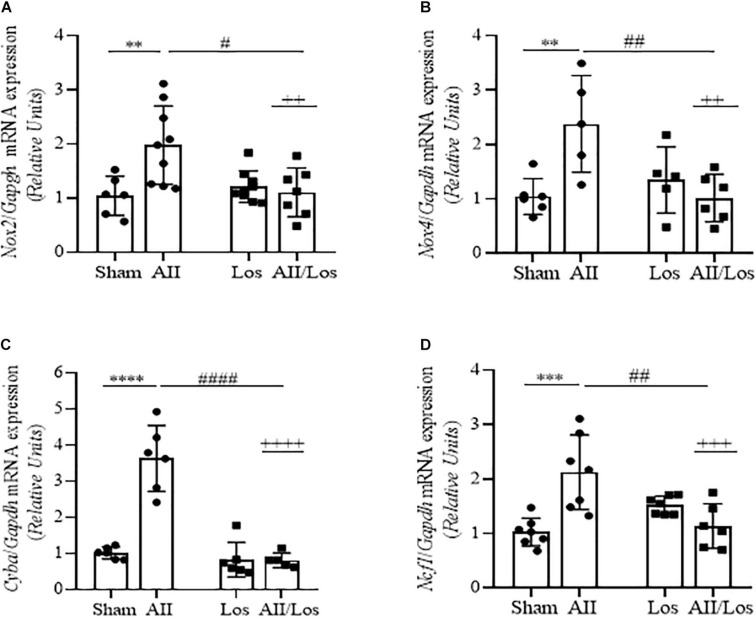
Effect of Ang II and/or losartan on NADPH oxidase subunit gene expression levels. *Nox2* **(A)**, *Nox4* **(B)**, *p22*^*phox*^ (*Cyba*; **C)**, and *p47*^*phox*^ (*Ncf1*; **D**), expression was analyzed in whole kidney tissue from the control rats and the rats treated with Ang II and/or losartan by quantitative PCR. The relative mRNA expression levels are expressed as the mean ± SD (*n* = 5–9 per group). The results were normalized to *Gapdh* expression (endogenous gene) and are shown as relative units compared to the sham (control) group; AII, angiotensin II, Los, losartan, AII/Los, and angiotensin II/losartan. ^++^*p* < 0.01, ^+++^*p* < 0.001, and ^++++^*p* < 0.0001 for the AII/Los interaction as indicated by two-way ANOVA. **p < 0.01, ***p < 0.001, and ****p < 0.0001 for AII *vs* sham or ^#^*p* < 0.05, ^##^*p* < 0.01, ^###^*p* < 0.001 and ^####^*p* < 0.0001 for AII/Los *vs* AII, as indicated by multiple comparisons.

To investigate whether prolonged treatment with Ang II/AT1R induced changes in oxidative stress factors, we measured the mRNA expression of NADPH oxidase subunits. As shown in Figures 1A–D and Table 2, in the whole kidney tissue of the Ang II-treated rats, the Nox2, Nox4, *Cyba* and *Ncf1* mRNA levels were significantly increased compared to those of the sham group rats, as demonstrated by multiple comparisons test. Losartan alone did not change these parameters compared to those in the sham group, but two-way ANOVA revealed a relevant interaction between Ang II and losartan in the cotreated group, since the stimulatory effect of Ang II was abolished by losartan treatment (interactions: Nox2, *p* = 0.008*;* Nox4, *p* = 0.0038*; Cyba*, *p* < 0.0001; and *Ncf1 p* = 0.0002).

**TABLE 2:** mRNA levels normalized to GAPDH.

Parameters		Multiple	Comparisons		Two-way AVOVA Interactions
mRNA expression	Sham (6–7)	AII (5–9)	Los (5–9)	AII/Los (5–8)	AII/Los
Nox2, *R.U*	1.04 ± 0.36	1.98 ± 0.73**	1.21 ± 0.29	1.11 ± 0.45^#^	*F* (1, 27) = 8.23^++^
		*p* = *0.0083*	*p* > *0.9999*	*p* > *0.0104*	*p* = *0.008*
Nox4, *R.U*	1.04 ± 0.33	2.38 ± 0.89**	1.35 ± 0.61	1.02 ± 0.43^##^	*F* (1, 18) = 11.06^++^
		*p* = *0.0084*	*p* > *0.9999*	*p* = *0.0071*	*p* = *0.0038*
*Cyba* (p22*^*phox*^*), *R.U*	1.01 ± 0.16	3.63 ± 0.91****	0.83 ± 0.48	0.81 ± 0.20^####^	*F* (1,19) = 33.44^++++^
		*p* < *0.0001*	*p* > *0.9999*	*p* < *0.0001*	*p* < *0.0001*
*Ncf1* (p47*^*phox*^*), *R.U*	1.03 ± 0.26	2.13 ± 0.68***	1.52 ± 0.17	1.14 ± 0.41^##^	*F* (1, 23) = 20.04^+++^
		*p* = *0.0005*	*p* = *0.2517*	*p* = *0.0024*	*p* = *0.0002*
*Atf4* (ATF4), *R.U*	1.01 ± 0.20	1.40 ± 0.31*	0.88 ± 0.13	0.83 ± 0.22^###^	*F* (1, 25) = 6.83^+++^
		*p* = *0.0188*	*p* > *0.9999*	*p* = *0.001*	*p* = *0.0002*
*Nfkb1* (NFkappaB), *R.U*	1.03 ± 0.27	1.56 ± 0.28**	1.09 ± 0.27	1.15 ± 0.16^#^	*F* (1, 25) = 6.81^+^
		*p* = *0.0040*	*p* > *0.9999*	*p* = *0.019*	*p* = *0.0151*
*Il1b* (IL1b), *R.U*	1.04 ± 0.26	1.69 ± 0.35**	0.86 ± 0.22	1.00 ± 0.17^##^	*F* (1, 16) = 4.67^+^
		*p* = *0.0071*	*p* > *0.9999*	*p* = *0.0047*	*p* = *0.0460*
*Acta2* (αSMA), *R.U*	0.97 ± 0.35	3.06 ± 1.39***	0.89 ± 0.29	0.96 ± 0.27^###^	*F* (1, 20) = 11.07^++^
		*p* = *0.0006*	*p* > *0.9999*	*p* = *0.0005*	*p* = *0.0034*

Two-way ANOVA with Bonferroni multiple comparisons test was performed to compare data (means ± SD). The number of animals per group is indicated in parentheses. For multiple comparisons: *p < 0.05, **p < 0.01, ***p < 0.001, and ****p < 0.0001 vs sham (control); ^#^*p* < 0.05, ^##^*p* < 0.01, ^###^*p* < 0.001, and ^####^*p* < 0.0001 *vs* AII; *F* values and significance of the two-way ANOVA considering the interactions between AII/Los were given. ns and ^+^ indicate non-significant and significant at ^+^*p* < 0.05, ^++^*p* < 0.01, ^+++^*p* < 0.001, and ^++++^*p* < 0.0001, respectively. The data were normalized by *Gapdh* (endogen gene) and means expressed as relative units (*R.U*) to the sham group; *p*, *p*-value.

### ER Stress Factors

**FIGURE 2:**
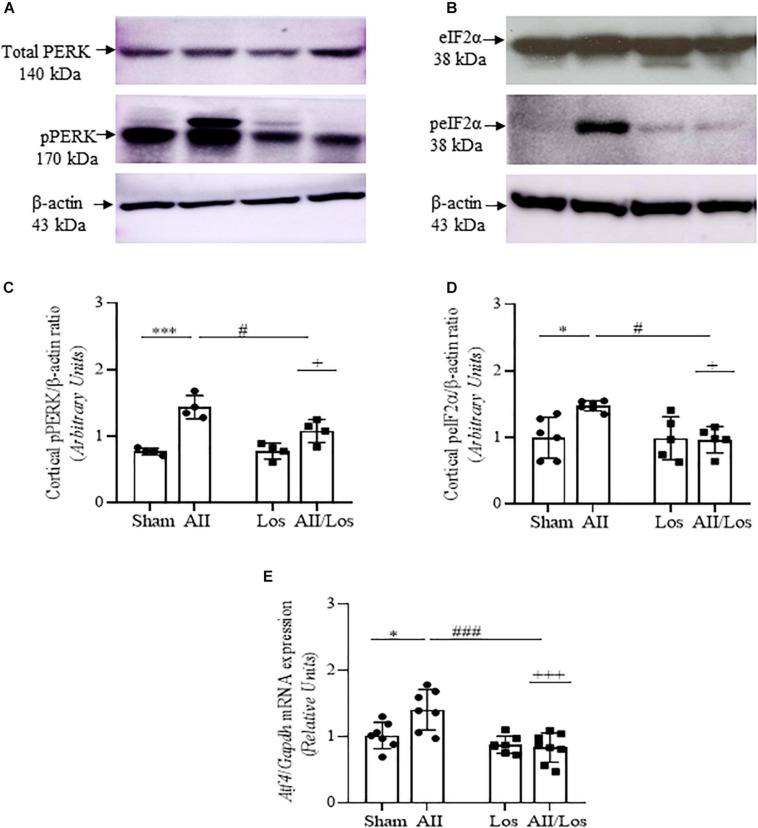
Effect of Ang II and/or losartan on the gene and protein expression levels of ER stress components. Whole kidney tissue or kidney cortex from the sham rats and the rats treated with Ang II and/or losartan were analyzed by quantitative PCR or immunoblotting, respectively. PERK **(A,B)** and eIF2α **(C,D)** protein expression (*n* = 4–8 per group) and *Atf4* mRNA expression levels (*n* = 5–9 per group; **E)**. For protein expression analysis, 50 μg samples of protein were resolved by 10 and 15% SDS-PAGE, and the data are expressed as the mean ± SD. β-actin was used as the loading control. For gene expression, the data are expressed as the mean ± SD and were normalized by *Gapdh*, an endogenous gene. Sham (control) group; AII, angiotensin II; Los, losartan; and AII/Los, angiotensin II/losartan.^ +^*p* < 0.05, ^+++^*p* < 0.001 for the AII/Los interaction, as indicated by two-way ANOVA. *p < 0.05, ***p < 0.001 for AII *vs* sham or ^#^*p* < 0.05, ^###^*p* < 0.001 for AII/Los *vs* AII, as indicated by multiple comparisons.

Next, we investigated whether prolonged treatment with Ang II/AT1R induced changes in ER stress factors (Figures 2A–E and Tables 2, 3). As shown by multiple comparisons test, Ang II treatment did not modify cortical non-phosphorylated PERK or eIF2α protein expression compared with that of the sham groups. In addition, Ang II treatment resulted in significant increases in cortical phosphorylated PERK and eIF2α protein expression, as well as whole-kidney tissue *Atf4* mRNA expression, compared to that of the sham groups. Losartan did not change these parameters compared to those of the sham groups. However, the significant interaction between Ang II and losartan for phosphorylated proteins and *Atf4* mRNA was shown by two-way ANOVA (interactions: pPERK, *p* = 0.0227, peIF2α, *p* = 0.0272; Atf4, *p* = 0.0002).

**TABLE 3:** Protein expression by western blotting and by immunofluorescence (Arbitrary Units).

Parameters		Multiple	Comparisons		Two-way ANOVA Interactions
Protein/β-actin	Sham (4–8)	AII (4–8)	Los (4–7)	AII/Los (5–7)	AII/Los (4–7)
Total PERK, *A.U*	1.02 ± 0.13	1.10 ± 0.33	1.09 ± 0.42	0.95 ± 0.04	*F* (1, 15) = 0.75^ ns^
		*p* > *0.9999*	*p* > *0.9999*	*p* > *0.9999*	*p* = *0.4001*
pPERKThr^980^, *A.U*	0.77 ± 0.05	1.44 ± 0.18***	0.78 ± 0.12	1.08 ± 0.17^#^	*F* (1, 12) = 6.82^+^
		*p* = *0.0001*	*p* > *0.9999*	*p* = *0.0209*	*P* = *0.0227*
Total eIF2α, *A.U*	1.04 ± 0.15	1.15 ± 0.06	1.09 ± 0.09	1.10 ± 0.04	*F* (1, 13) = 1.25^*ns*^
		*p* = *0.6372*	*p* > *0.9999*	*p* > *0.9999*	*p* = *0.2836*
peIF2α Ser^51^, *A.U*	1.00 ± 0.30	1.47 ± 0.07*	0.99 ± 0.32	0.96 ± 0.19^#^	*F* (1, 18) = 5.78^+^
		*p* = *0.0200*	*p* > *0.9999*	*p* = *0.0175*	*p* = *0.0272*
αSMA, *A.U*	1.03 ± 0.09	1.66 ± 0.37**	1.06 ± 0.13	0.86 ± 0.26^###^	*F* (1, 17) = 17.06^+++^
		*p* = *0.0151*	*p* > *0.9999*	*p* = *0.0027*	*p* = *0.0007*
Cortex NHE3, *A.U*	1.00 ± 0.21	1.78 ± 0.34***	1.03 ± 0.17	1.26 ± 0.29^#^	*F* (1, 21) = 7.15^+^
		*p* = *0.0001*	*p* > *0.9999*	*p* = *0.0170*	*p* = *0.0142*
Cortex *A.U*	1.00 ± 0.27	1.47 ± 0.23*	0.78 ± 0.23	1.10 ± 0.29	*F* (1, 18) = 0.48^*ns*^
		*p* = *0.0473*	*p* > *0.9999*	*p* = *0.1921*	*p* = *0.4975*
Cortex NHE1, *A.U*	0.99 ± 0.16	2.15 ± 0.85***	1.02 ± 0.16	1.13 ± 0.42^##^	*F* (1, 26) = 8.05^++^
		*p* = *0.0060*	*p* > *0.9999*	*p* = *0.0033*	*p* = *0.0087*
Cortex ENaC β, *A.U*	1.00 ± 0.11	1.62 ± 0.37**	0.93 ± 0.29	1.02 ± 0.37^#^	*F* (1, 21) = 4.88^+^
		*p* = *0.0080*	*p* > *0.9999*	*p* = *0.0144*	*p* = *0.0384*
Cortex NKCC2, *A.U*	1.00 ± 0.14	1.73 ± 0.33**	0.72 ± 0.41	0.68 ± 0.27^###^	*F* (1, 21) = 8.72^++^
		*p* = *0.0031*	*p* > *0.9999*	*p* = *0.0001*	*p* = *0.0080*
Cortex NCC, *A.U*	0.85 ± 0.18	1.35 ± 0.20**	0.86 ± 0.22	0.88 ± 0.18^#^	*F* (1, 16) = 7.28^+^
		*p* = *0.0072*	*p* > *0.9999*	*p* = *0.0125*	*p* = *0.0158*

**Protein FI**	**Sham (5)**	**AII (6–7)**	**Los (5)**	**AII/Los (6)**	**AII/Los (6)**

NKCC2, *A.U*	2.19 ± 0.30	3.99 ± 0.99***	1.43 ± 0.19	1.78 ± 0.36^####^	*F* (1, 18) = 8.60^++^
		*p* = *0.0004*	*p* = *0.3111*	*P* < *0.0001*	*p* = *0.0089*
NCC, *A.U*	2.67 ± 0.58	3.90 ± 0.77**	1.87 ± 0.32	2.09 ± 0.23^####^	*F* (1, 19) = 4.70^+^
		*p* = *0.0066*	*p* = *0.1852*	*p* < *0.0001*	*p* = *0.0429*

Two-way ANOVA with Bonferroni multiple comparisons test performed to compare data (means ± SD). The number of animals per group is indicated in parentheses. For multiple comparisons: *p < 0.05, **p < 0.01, and ***p < 0.001 *vs* sham (control); ^#^*p* < 0.05, ^##^*p* < 0.01, ^###^*p* < 0.001, and ^####^*p* < 0.0001 *vs* angiotensin II (AII); *F* values and significance of the two-way ANOVA considering the treatments with angiotensin AII (AII), Losartan (Los), and their interactions were given. ns and ^+^ indicate non-significant and significant at ^+^*p* < 0.05, ^++^*p* < 0.01, and ^+++^*p* < 0.001, respectively. The values were normalized by β-actin (loading control) and means expressed as arbitrary units (*A.U*) to the control group; FI (Fluorescence Intensity); *p*, *p*-value.

### Proinflammatory and Profibrotic Factors

**FIGURE 3:**
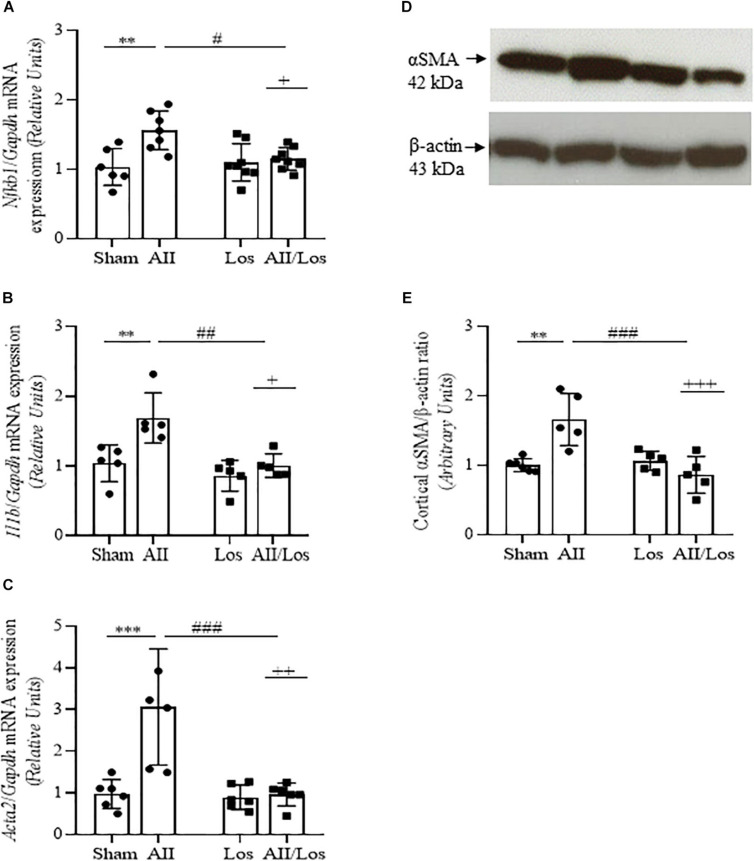
Effect of Ang II and/or losartan on the gene and protein expression levels of proinflammatory and profibrotic components. Whole kidney tissue or kidney cortex from the sham rats and the rats treated with Ang II and/or losartan were analyzed by quantitative PCR or immunoblotting, respectively. *Nfkb1* **(A)**, *Il1b* **(B)**, and *Acta2* (αSMA) mRNA expression (**C**; *n* = 5–9 per group) and αSMA protein expression (**D,E**; *n* = 4–8 per group). For gene expression, the data are expressed as the mean ± SD and were normalized by *Gapdh*, an endogenous gene. For protein expression analysis, 50 μg samples of protein were resolved by 15% SDS-PAGE, the data are expressed as the mean ± SD, and β-actin was used as the loading control. Sham (control) group; AII, angiotensin II; Los, losartan; and AII/Los, angiotensin II/losartan. ^+^*p* < 0.05, ^++^*p* < 0.01, and ^+++^*p* < 0.001 for the AII/Los interaction, as indicated by two-way ANOVA. **p < 0.01, ***p < 0.001 for AII *vs* sham or ^#^*p* < 0.05, ^##^*p* < 0.01, and ^###^*p* < 0.001 for AII/Los *vs* AII, as indicated by multiple comparisons.

Using multiple comparisons tests, as shown in Figure 3 and Tables 2, 3, we found that prolonged Ang II treatment resulted in increased *Nkfb1, Il1b, and Acta2* mRNA expression, as well as αSMA protein expression, compared to that of the sham groups. The rats in the sham or losartan-treated groups did not exhibit any changes in these parameters. However, in the cotreated group, losartan inhibited the stimulatory effect of Ang II on these parameters. This effect was confirmed by two-way ANOVA, in which interactions were relevant (mRNA: *Nfkb1*, *p* = 0.0151*;* Il1b, *p* = 0.0460 and Acta2, *p* = 0.0034 mRNA and α-SMA protein, *p* = 0.0007).

### Na^+^ Transporters

**FIGURE 4:**
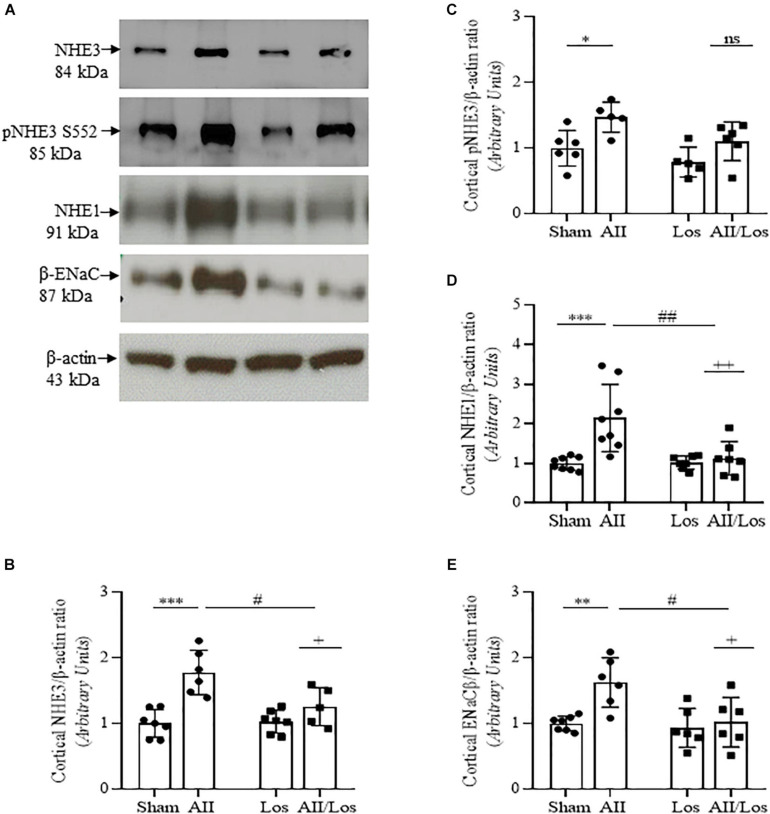
Effect of Ang II and/or losartan on the protein expression levels of kidney Na^+^ transporters. Protein expression in kidney cortex tissue from sham rats and the rats treated with Ang II and/or losartan. Representative immunoblots showing total Na^+^/H^+^ exchanger isoform 3 (NHE3), phospho/S552/NHE3, Na^+^/H^+^ exchanger isoform 1 (NHE1) and epithelial Na^+^ channel subunit beta (ENaCβ; **A**). For immunoblot analysis, 50 μg samples of protein were resolved by 10% SDS-PAGE. The values are expressed as the mean ± SD (*n* = 5–8 per group), and β-actin was used as a loading control. The quantification of bands is represented as total NHE3 **(B)**, phospho-S552 NHE3 **(C)**, NHE1 **(D)**, and ENaC β **(E)**. Sham (control) group; AII, angiotensin II, Los, losartan; and AII/Los, angiotensin II/losartan. ^+^*p* < 0.05,^ ++^*p* < 0.01, and ^+++^*p* < 0.001 for the AII/Los interaction, as indicated by two-way ANOVA. *p < 0.0, **p < 0.01, and ***p < 0.001 for AII *vs* sham or ^#^*p* < 0.05, ^##^*p* < 0.01, and ^###^*p* < 0.001 for AII/Los *vs* AII, as indicated by multiple comparisons.

Considering that Ang II and oxidative stress induce changes in renal salt and water reabsorption, the prolonged effect of Ang II on kidney cortex Na^+^ transporters was evaluated. Using multiple comparisons tests, we revealed that Ang II treatment resulted in increased protein expression of cortical Na^+^/H^+^ exchanger 3 (NHE3) and increased phospho/S552/NHE3 protein expression, the latter indicating reduced activity of NHE3, as shown in Figures 4A–E and Table 3 (Kocinsky et al., 2007). Moreover, the rats in the Ang II-treated group displayed robust increases in Na^+^/H^+^ exchanger 1 (NHE1) and epithelial Na^+^ channel (ENaC, subunit β) protein expression compared to the sham groups. The rats treated with losartan did not exhibit differences in the expression of the above-mentioned proteins, while the rats in the Ang II/losartan group displayed significant decreases in protein expression compared to the Ang II-treated rats. Most of these observations were confirmed by two-way ANOVA, in which interactions were shown for cortical NHE3 (*p* = 0.0142), NHE1, (*p* = 0.0087), and ENaC β (*p* = 0.0384) but not cortical pS552NHE3 (*p* = 0.4975).

**FIGURE 5:**
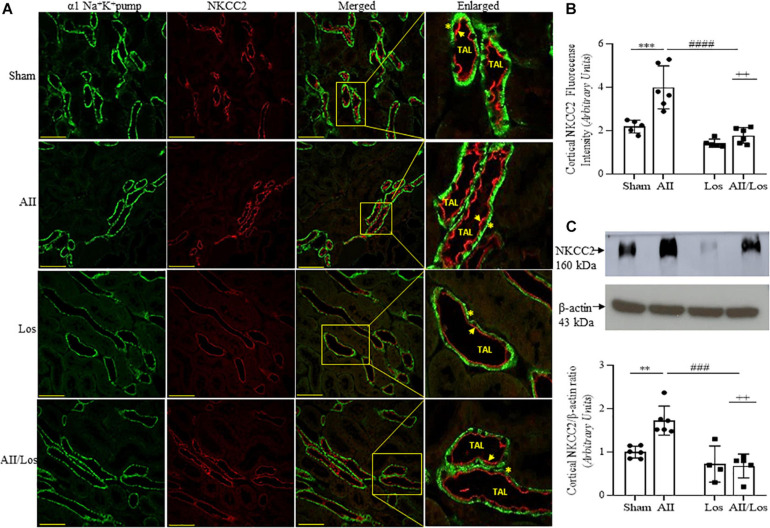
Effect of Ang II and/or losartan on immunofluorescence and protein expression levels of kidney cortical Na-K-2Cl cotransporter (NKCC2). Immunofluorescence images showing NKCC2 staining in the cortical area (indicated by arrows), and the basolateral α1Na^+^K^+^ pump is indicated by asterisks in the thin ascending limb (TAL). Images were captured using a Zeiss LSM 510 confocal microscope equipped with a 40 × objective. α1Na^+^K^+^ pump staining was used as the basolateral membrane integrity control, and NKCC2 and α1Na^–^ K pump staining were merged. The enlarged images are also shown. Bar = 50 μm **(A)**. The mean fluorescence intensity of NKCC2 is also presented (**B**; *n* = 5–7 per group). For immunoblot analysis, 50 μg cortical protein samples were resolved by 9% SDS-PAGE. The values are expressed as the mean ± SEM (*n* = 4–6 per group), and β-actin was used as the loading control **(C)**. Sham (control) group; AII, angiotensin II; Los, losartan; and AII/Los, angiotensin II/losartan.^ ++^*p* < 0.01 for the Ang II and losartan interaction, as indicated by two-way ANOVA. **p < 0.01, ***p < 0.001 for Ang II *vs* sham or ^#^*p* < 0.05, ^####^*p* < 0.0001 for AII/Los *vs* AII, as indicated by multiple comparisons.

Next, we investigated cortical Na-K-2Cl (NKCC2) and Na-2Cl (NCC) protein expression and distribution. According to multiple comparisons tests, in the rats with prolonged Ang II treatment, cortical NKCC2 staining (red) in the luminal surface of the thin ascending limb of the long loop (TAL), as well as cortical protein expression levels evaluated by immunoblotting, were significantly higher than those of the sham groups. In the losartan-treated group, protein staining and expression seemed to be normal compared to that in the sham group, but losartan treatment significantly attenuated the stimulatory effect of Ang II on protein staining and expression levels (Figures 5A–C and Table 3). In this last group, the interaction between Ang II and losartan was confirmed by two-way ANOVA (WB: NKCC2, *p* = 0.0080, stained: NKCC2, *p* = 0.0089).

**FIGURE 6:**
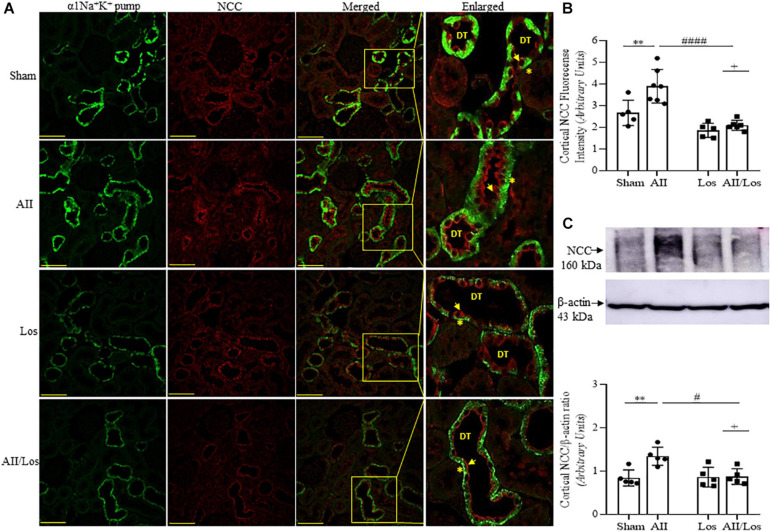
Effect of Ang II and/or losartan on immunofluorescence and protein expression levels of kidney cortical Na-2Cl cotransporter (NCC). Immunofluorescence images showing NCC staining in the cortical area (indicated by arrows), and the basolateral α1Na^+^K^+^ pump is indicated by asterisks in the distal tubule (DT). Images were captured using a Zeiss LSM 510 confocal microscope equipped with a 40 × objective. α1Na^+^K^+^ pump staining was used as the basolateral membrane integrity control, and NCC and α1Na^–^ K pump staining were merged. The enlarged images are also shown. Bar = 50 μm **(A)**. The mean fluorescence intensity of NCC is also presented (**B**; *n* = 5–7 per group). For immunoblot analysis, 50 μg cortical protein samples were resolved by 9% SDS-PAGE. The values are expressed as the mean ± SEM (*n* = 4–6 per group), and β-actin was used as the loading control **(C)**. Sham (control) group; AII, angiotensin II; Los, losartan; and AII/Los, angiotensin II/losartan. ^+^*p* < 0.05 for the Ang II and losartan interaction, as indicated by two-way ANOVA. **p < 0.01 for Ang II *vs* sham or ^#^*p* < 0.05, ^####^*p* < 0.0001 for AII/Los *vs* AII, as indicated by multiple comparisons.

Immunoblot analysis by multiple comparisons test showed that prolonged Ang II treatment also increased cortical NCC staining (red) on the luminal surface of the convoluted distal tubule and cortical protein expression compared to those of the sham group. The losartan-treated groups did not exhibit changes in the protein expression levels, but the stimulatory effect of Ang II on this parameter was impaired, and the interaction between Ang II and losartan was confirmed by two-way ANOVA (WB: NCC, *p* = 0.0158, stained: NCC, *p* = 0.0429; Figures 6A–C and Table 3). For all immunofluorescence analyses, epithelial polarity was confirmed by α1Na^+^K^+^ pump staining (green) on the basolateral membrane.

## Discussion

In scientific and clinical contexts, the association between hypertension and renal disease has been confirmed. However, it has been difficult to quantify the contribution of hypertension to progressive renal disease, since the major aspects involved remain poorly understood. In addition, during episodic or sustained increases in systemic blood pressure, the preglomerular microvasculature sustains renal autoregulatory responses, providing primary protection against hypertensive renal damage, since the mean blood pressure remains below an acceptable limit and within the autoregulatory range (Bidani and Griffin, 2004).

However, Ang II-mediated hypertension and tissue damage have mainly been investigated with regard to vascular smooth muscle cell hypertrophy and hyperplasia in systemic blood vessels (Long et al., 2004; Mennuni et al., 2014), but in this condition, the effects of Ang II in the kidney as well the cellular and molecular mechanisms have been poorly characterized. From a therapeutic point of view, the pathology of hypertensive renal disease can be attenuated by reduction of systemic and preglomerular blood pressure load, as well as regulation of cellular and molecular pathways that mediate tissue injury (Bidani and Griffin, 2004). In this context, AT1R antagonists are important antihypertensive agents mainly when combined with diuretics such as hydrochlorothiazide (HCTZ) or angiotensin converting enzyme (ACE) inhibitors (Ueda et al., 2012; Perini et al., 2020). Thus, investigating the effects of AT1R antagonists on the deleterious intrarenal effects induced by Ang II seems to be relevant for scientific and clinical practice.

Given the relevance of Ang II/AT1R-mediated hypertension and kidney tissue injury observed in our previous study (Casare et al., 2016), using the same experimental model, we investigated whether these deleterious effects of Ang II/AT1R on kidney function and morphology are related to oxidative stress and ER stress. In the kidney, Ang II has been shown to stimulate the production of superoxide anion via NADPH oxidase activity mediated by arachidonic acid (Block et al., 2006), PKC (Jaimes et al., 1998), Rac1 (Gorin et al., 2003), and Erk1/2 (Gorin et al., 2004) signaling. In addition, superoxide anion leads to vascular dysfunction, glomerular injury and proteinuria (Araujo and Wilcox, 2014). Other findings suggest that the NOX family of proteins requires the chaperone protein p22^*phox*^ for the activation of several cytoplasmic subunits, including p47^*phox*^ (Gill and Wilcox, 2006). Indeed, the interaction of NOX4 with p22*^*phox*^* seems to be related to fibroblast-to-myofibroblast differentiation and consequent kidney fibrosis, as reported by other studies of diabetic models (Gorin et al., 2005; Bondi et al., 2010). Additionally, our current study revealed a pronounced stimulatory effect of Ang II mediated by AT1R on the transcription of different NADPH oxidase subunits, including NOX2, NOX4, *Cyba* (p22*^*phox*^*) and *Ncf1* (p47*^*phox*^*), suggesting the source of renal oxidative stress under prolonged high levels of circulating Ang II.

Reactive oxygen species can be produced in the cytosol and in several organelles, including the ER and mitochondria (Cao and Kaufman, 2014). Indeed, it has been observed that under diabetic conditions, ROS accumulation can induce DNA and protein damage, resulting in ER stress, cell injury, and CKD progression (Henriksen et al., 2011). Under physiological conditions, the ER is the site of protein synthesis and folding, and these functions are modulated by glucose-regulated protein (GRP78), which facilitates polypeptide folding and assembly, prevents misfolding and aggregation and acts as a mediator of the unfolded protein response (UPR; Ron and Walter, 2007), which involves the stress sensors protein kinase R (PKR)-like ER kinase (PERK), inositol-requiring enzyme 1 (IRE1), and activating transcription factor 6 (ATF6; Gonçalves et al., 2018). However, under ER stress conditions, misfolded proteins recruit the ER stress protein GRP78 to restore proper protein folding. Then, free sensor proteins are autoactivated to phosphorylate other proteins associated with ER stress responses (Gorostizaga et al., 2013). We previously reported robust expression of GRP78 in the injured kidneys of rats subjected to prolonged Ang II treatment (Cardoso et al., 2018). In addition to these findings, the present study revealed that the prolonged effect of Ang II/AT1R on the ER stress response was mediated by increased PERK and eIF2α protein expression and *Atf4* mRNA expression and increased eIF2α protein expression. Taken together, our results indicate that the GRP78/PERK/eIF2α/ATF4 pathway enhances the progression of Ang II/AT1R/ROS-mediated chronic kidney injury. However, we did not discount the possibility that Ang II/AT1R signaling induces ER stress and indirectly induces ROS production because the NADPH pool is preserved at the ER lumen, as previously reported (Piccirella et al., 2006).

Accumulating evidence demonstrates that the combination of oxidative stress, ER stress and inflammation amplifies the progression of many diseases (Dandekar et al., 2015). In addition, the phosphorylation of eIF2α and/or PERK signaling results in increased ATF4 transcription, followed by NFkappaB1 activation in several organs, including the kidneys (Scheidegger et al., 1999; Ruiz-Ortega et al., 2001; Brown and Naidoo, 2012). NFkappaB1 can induce the transcription of proinflammatory cytokines, such as interleukin-1β (IL-1β) and chemokines, in addition to adhesion molecules and superoxide anions (Wang et al., 2014). In turn, IL-1β can activate NFkappaB1/p65 complex (Shi and Berger, 2018) to sustain the inflammatory cycle (Anders, 2016) and induce α-SMA expression in glomerular cells (Wang et al., 2002). Notably, α-SMA is normally expressed by vascular smooth muscle cells, but interstitial fibroblasts and mesangial and tubular cells can also express this protein in a variety of glomerular diseases, which are frequently associated with increased cell proliferation and increased extracellular matrix production (Geleilete et al., 2001). Indeed, we previously observed renovascular remodeling, glomerular expansion and glomerulosclerosis in the same experimental model (Casare et al., 2016). Thus, in the current study, our data are consistent with these findings, since the chronic effect of Ang II/AT1R resulted in increased *Nfkb1, Il1b and Acta2* (α-SMA) gene expression, as well as α-SMA protein expression. Together, these results indicate important integration between Ang II/AT1R/ROS/ER stress/NFkappaB1 signaling and proinflammatory and profibrotic signaling, which ultimately can mediate chronic kidney disease progression.

Kidney function is essential to the maintenance of internal volume and blood pressure, mainly through tubular fluid and electrolyte reabsorption. In this context, the activity of Na^+^ transporters, such as Na^+^/H^+^ exchanger 1 (NHE1) and 3 (NHE3), the cotransporters Na-K-2Cl (NKCC2) and Na-Cl (NCC), and Na^+^ channels (ENaC, subunits α, β, and γ), is highly relevant. In addition to regulating internal volume, NHE1 is the most important isoform associated with intracellular pH homeostasis and is involved in the cell cycle and resistance to apoptosis (Wu et al., 2004; Costa-Pessoa et al., 2014). However, it has been established that the effect of Ang II on salt and water processing by the kidney occurs in a dose- and time-dependent manner. A modest increase in circulating Ang II levels is enough to stimulate Na^+^ reabsorption in the nephron via protein kinase C (PKC) activation (Gonzalez-Vicente and Garvin, 2017). In addition, Gonzalez-Vicente et al. (2016) used acute Ang II-treated rats reported a positive link among high levels of plasma Ang II, ROS production and increased NHE3 activity. Nguyen et al. (2015) reported that Ang II infusion (200 ng/kg/min) in rats for 72 h resulted in prehypertension associated with increased protein expression of NHE3, NKCC2, NCC, and cortical ENaC subunits. Rats treated with Ang II (400 ng/kg/min) for 2 weeks also exhibited hypertension but without changes in NKCC2 or ENaC alpha and beta protein expression. However, a significant increase in NCC protein expression was noted. Other studies showed that adult mice treated with Ang II (800 ng/kg/min) for 7 days developed severe hypertension and had increased NHE3 and NCC expression and decreased NKCC2 protein expression (Tiwari et al., 2009, 2010).

Considering this lack of consistency in Ang II-induced Na^+^ transporter protein expression, as well as the contribution of AT1R to this process, in the present study, we investigated whether prolonged Ang II infusion over 2 weeks and/or losartan treatment could modulate cortical Na^+^ transporters expression. Our results indicate that although Ang II/AT1R stimulates the total and phosphorylated NHE3 at serine 552, consistent with a discrete inactivation and anchoring of NHE3 at the base of the microvillus (Nguyen et al., 2015), with two-way ANOVA, the interaction between Ang II/losartan was confirmed by non-phosphorylated, but not by the phosphorylated form. It is known that pressor doses of Ang II acutely induce internalization of NHE3 in the proximal tubule and natriuresis (Yang et al., 2002), whereas long-term subpressor doses of Ang II increase the expression of total NHE3 and elevate blood pressure (Banday and Lokhandwala, 2011), consistent with Ang II-induced cAMP/PKA signaling downregulation and consequent decreases of NHE3 phosphorylation at serine 552 (Girardi and Di Sole, 2012). In addition, Banday and Lokhandwala (2011) reported that AT1R stimulation under oxidative stress conditions, results in NHE3 activation in proximal tubule via Ca^2+^-calmodulin- dependent mechanism. These observations were consistent with our results, since the long-term pressor dose of Ang II/AT1R was more effective to increases the expression of total NHE3, compared to the phosphorylated form.

It is important to emphasize that our experimental model has increased GFR and tubular flow, condition that results in sodium overload in the posterior segments of the nephron (Casare et al., 2016). Indeed, our results revealed a significant increase in renal cortex NKCC2, NCC, and ENaC β protein expression, and losartan inhibited the stimulatory effect of Ang II on these transporters. Taken together, these data suggest that despite NHE3 stimulation, the increased tubular flow and sodium overload seems to be determinant conditions to amplify the capacity of the cortical nephron for Na^+^ reabsorption and diuresis. For note, the internal electrolyte and water content was in balance. Interestingly, in our experimental model, Ang II/AT1R induced an increase in NHE1 protein expression, suggesting the contribution of this protein to the proliferative responses of the renal microvasculature and mesangial cells.

## Conclusion

Chronic kidney pathologies are related to different factors and cellular programming pathways, and these responses involve risks that can lead to the complete loss of kidney function. The major findings of our study are that in chronic Ang II-mediated hypertension, kidney tissue expresses signals for relevant biomarkers of oxidative stress and ER stress, which are closely related to the progression of kidney damage and changes in tubular sodium handling. Prolonged Ang II/AT1R effect over 2 weeks results in increased Na^+^ reabsorption, which seems to be repaired by increased urinary flow and Na^+^ overload. Finally, our findings also highlight the therapeutic potential of losartan, which, in addition to its antihypertensive effect, plays an important role in kidney tissue, reducing oxidative stress and ER stress signals and recovering relevant parameters of the maintenance of renal function, preventing the progression of Ang II-induced CKD.

## Data Availability Statement

The raw data supporting the conclusions of this article will be made available by the authors, without undue reservation.

## Ethics Statement

The experimental protocols were reviewed, approved and conduced in accordance with the Ethics Committee on the Use of Experimental Animals of the University of São Paulo (No. 37/2016) and were in accordance with the ethical principles adopted by the Brazilian Society of Laboratory Animal Science.

## Author Contributions

BL designed and performed all experiments, analyzed the data, and helped write and review the manuscript. FC and FF helped with the animal treatments. GG contributed to the immunofluorescence/immunoblotting experiments. MO-S supported and supervised the study and contributed to the writing and review of the manuscript. All authors approved the final manuscript.

## Conflict of Interest

The authors declare that the research was conducted in the absence of any commercial or financial relationships that could be construed as a potential conflict of interest.
